# Academic performance data of undergraduate students׳ in 23 programmes from a private University in Nigeria

**DOI:** 10.1016/j.dib.2018.07.056

**Published:** 2018-07-27

**Authors:** Azubuike Ezenwoke, Oluwadamilola Ogunwale, Opeyemi Matiluko, Emmanuel Igbekele, Samuel Dare, Omotola Ezenwoke, Adeniyi Olayanju

**Affiliations:** a Department of Computer and Information Sciences, Covenant University, Ota, Nigeria; b Department of Economics, Ajayi Crowther University, Oyo, Nigeria; c Centre for Systems and Information Services, Landmark University, Omu-aran, Nigeria; d Academic Office, Landmark University, Omu-aran, Nigeria; e Department of Accounting and Finance, Landmark University, Omu-aran, Nigeria; f Department of Agricultural and Bio-systems Engineering, Landmark University, Omu-aran, Nigeria

## Abstract

The quality of teaching and learning in higher education in many developing countries can be improved as institutions in this region adopt evidence-based practices that emphasize empirical measurements, observations, analysis and reports of learning outcomes. This article presents and analyses data on the academic performances of undergraduate students for duration of three semesters across the three major colleges of Landmark University, a private University in Nigeria. The colleges include the college of Agricultural Sciences (CAS), college of Business and Social Sciences (CBSS), and the college of Science and Engineering (CSE). Furthermore, population samples of 82, 577 and 812 undergraduates were selected randomly from CAS, CBSS and CSE respectively; totaling a population of sample of 1471 undergraduates from all academic levels (200L–500L) with the exception of first year students. The random selection was drawn from three consecutive semesters- the first and second semesters of academic 2016/2017 session and first semester of 2017/2018 academic session. The cumulative GPA of the sample population of students for the semester highlighted was obtained from the Centre for Systems and Information Services Units of the University. Motivated by the need to promote evidence-based research in academic excellence, a spread-sheet containing the detailed dataset is attached to this article. The descriptive statistics and frequency distributions of academic performance data are presented in with the use of tables and graphs for easy data interpretations. The data provided in this article supports the goal of a regional policy towards the realization of qualitative sustainable education.

**Specifications Table**

**Table:** 

**Subject area**	Agricultural Sciences, Business and Social Sciences and Sciences and Engineering Education
**Specific area of interest**	Analysis of Academic Performance Data
**Data type**	Tables, graphs and spread-sheet file
**Data collection**	Academic performance data comprising Grade Point Average (GPA) for a three semester period for multi-level undergraduate students studying programmes relating to Agricultural sciences. Sciences and Engineering, and Business and Social Sciences in a private University in Nigeria. The data was obtained from the Centre for Systems and Information Services of the university.
**Data layout**	Raw, grouped
**Experimental factors**	First year undergraduate students were excluded
**Experimental structures**	Descriptive statistics and frequency distributions are performed to show the distribution of the academic performance across the three colleges, various programmes and different levels.
**Data source location**	The population sample and the information on academic performance provided in this article were obtained at Landmark University, Omu-Aran, Nigeria
**Accessibility of data**	Detailed datasets in a Microsoft Excel spread-sheet file attached to this article are made publicly available.

**Value of the data**

• Comprehensive datasets on academic outcomes encourages confidence in evidence-based research to understand factors affecting academic excellence and skills acquisition in developing countries.
• The accessibility of academic outcomes dataset will foster the attainment of sustainable education and formulation of practicable regional policies geared towards improving teaching and learning pedagogies.
• The field of learning analytics, together with advances in data mining, machine learning and data analytics will benefit from the availability of empirical academic performance data for developing predictive models to studying outcomes in undergraduate programmes.
• Statistical analysis such as descriptive statistics and frequency distribution, presented in tabular and graphical-forms simplifies data interpretation in other to draw useful deductions and reasonable conclusions.

## 1 Data

The quality of teaching and learning in higher education in many developing countries can be improved as institutions in this region adopt evidence-based practices that emphasize empirical measurements, observations, analysis and reports of learning outcomes [1], [2], [3]. This article presents and analyses data on the academic performances of undergraduate students of Landmark University, a Nigerian private University. Landmark University is a private faith-based University located in Omu-Aran, Kwara State in Nigeria.

The data comprise academic performance index for the duration of three semesters across the three major colleges of the university. The colleges include the college of Agricultural Sciences (CAS), college of Business and Social Sciences (CBSS), and the college of Science and Engineering (CSE). Furthermore, population samples of 82, 577 and 812 undergraduates were selected randomly from CAS, CBS and CSE respectively; totaling a population of sample of 1471 undergraduates from all academic levels (200L–500L) with the except of first year students. The random selection was drawn from three consecutive semesters- the first and second semesters of academic 2016/2017 session and first semester of 2017/2018 academic session.

However, the process of selection excluded undergraduates with incomplete academic records. A total of 2, 5 and 222 undergraduates were pooled from CAS, CBSS, and CSE respectively. Table 1, Table 2, Table 3, Table 4, Table 5, Table 6, Table 7, Table 8, Table 9, Table 10, Table 11, Table 12, Table 13, Table 14, Table 15, Table 16, Table 17, Table 18, Table 19, Table 20, Table 21, Table 22, Table 23 contains the descriptive statistics of the academic performances of undergraduates in the twenty-two programmes offered at Landmark University.

**Table 1:** Descriptive statistics of academic performances of undergraduates studying Agricultural Economics.

	Grade Point Average (GPA)			
	First semester	Second semester	First semester	Aggregate
	(2017)	(2016)	(2016)	
Mean	3.171828	3.857424	3.89464	3.641297
Standard Error	0.250804	0.181723	0.226964	0.199028
Median	3.2857	4.1154	4.2222	3.9925
Mode	–	–	5	–
Standard Deviation	1.254018	0.908617	1.134818	0.995138
Sample Variance	1.572561	0.825585	1.287811	0.990299
Kurtosis	− 0.62466	− 0.59205	− 1.31708	− 0.7888
Skewness	− 0.44565	− 0.74602	− 0.57434	− 0.63987
Range	4.3636	2.9333	3	3.345133
Minimum	0.6364	2	2	1.585
Maximum	5	4.9333	5	4.930133
Sum	79.2957	96.4356	97.366	91.03243
Total samples	25	25	25	25

**Table 2:** Descriptive statistics of academic performances of undergraduates studying Agricultural Extension and Rural Development.

	Grade Point Average (GPA)			
	First semester	Second semester	First semester	Aggregate
	(2017)	(2016)	(2016)	
Mean	3.817339	3.978667	3.976594	3.727426
Standard Error	0.142425	0.119774	0.149272	0.047632
Median	3.95835	4.0316	4	3.764839
Mode	–	4.2692	3.8333	–
Standard Deviation	0.604257	0.508158	0.633306	0.202087
Sample Variance	0.365127	0.258224	0.401077	0.040839
Kurtosis	− 0.58994	− 0.5932	− 0.85297	− 0.74922
Skewness	− 0.57423	− 0.51472	− 0.02345	− 0.67057
Range	2.0417	1.7769	2.0323	0.602507
Minimum	2.7083	2.9231	2.9677	3.344843
Maximum	4.75	4.7	5	3.94735
Sum	68.7121	71.616	71.5787	67.09367
Total samples	18	18	18	18

**Table 3:** Descriptive statistics of academic performances of undergraduates studying Animal Science.

	Grade Point Average (GPA)			
	First semester	Second semester	First semester	Aggregate
	(2017)	(2016)	(2016)	
Mean	3.283339	3.724879	3.512782	3.639958
Standard Error	0.241285	0.135938	0.219568	0.086089
Median	3.375	3.7628	3.45835	3.689819
Mode	3.9167	3.9231	5	–
Standard Deviation	1.276759	0.719317	1.161845	0.455539
Sample Variance	1.630112	0.517416	1.349883	0.207516
Kurtosis	− 0.49587	− 1.20335	− 0.83309	− 1.56994
Skewness	− 0.67226	− 0.04327	− 0.32604	− 0.00454
Range	4.375	2.4205	3.8333	1.28313
Minimum	0.5	2.3462	1.1667	2.988324
Maximum	4.875	4.7667	5	4.271454
Sum	91.9335	104.2966	98.3579	101.9188
Total samples	28	28	28	28

**Table 4:** Descriptive statistics of academic performances of undergraduates studying Crop Science.

	Grade Point Average (GPA)			
	First semester	Second semester	First semester	Aggregate
	(2017)	(2016)	(2016)	
Mean	3.722222	3.903444	3.887056	4.317909
Standard Error	0.323493	0.196498	0.225995	0.028217
Median	4.0833	3.8462	4.2	4.274217
Mode	4.0833	–	–	–
Standard Deviation	0.970479	0.589493	0.677985	0.084652
Sample Variance	0.94183	0.347502	0.459664	0.007166
Kurtosis	− 0.15111	− 1.10061	− 1.43487	− 0.17437
Skewness	− 1.05305	− 0.2114	− 0.59243	1.248455
Range	2.75	1.7308	1.8	0.209331
Minimum	1.875	3	2.8	4.258465
Maximum	4.625	4.7308	4.6	4.467796
Sum	33.5	35.131	34.9835	38.86119
Total samples	9	9	9	9

**Table 5:** Descriptive statistics of academic performances of undergraduates studying Soil Science.

	Grade Point Average(GPA)			
	First semester	Second semester	First semester	Aggregate
	(2017)	(2016)	(2016)	
Mean	4.0238	4.63335	5	4.512561
Standard Error	0.2619	0.13335	0	0.012476
Median	4.0238	4.63335	5	4.512561
Mode	–	–	5	–
Standard Deviation	0.370383	0.188585	0	0.017644
Sample Variance	0.137183	0.035564	0	0.000311
Kurtosis	–	–	–	–
Skewness	–	–	–	–
Range	0.5238	0.2667	0	0.024952
Minimum	3.7619	4.5	5	4.500085
Maximum	4.2857	4.7667	5	4.525037
Sum	8.0476	9.2667	10	9.025122
Total samples	2	2	2	2

**Table 6:** Descriptive statistics of academic performances of undergraduates studying Accounting.

	Grade Point Average (GPA)			
	First semester	Second semester	First semester	Aggregate
	(2017)	(2016)	(2016)	
Mean	3.541968	3.67936	3.570868	3.514568
Standard Error	0.083408	0.074883	0.076197	0.046316
Median	3.7826	3.84105	3.625	3.537235
Mode	4.6667	4.1579	4.8	–
Standard Deviation	1.028325	0.923223	0.939414	0.571025
Sample Variance	1.057453	0.85234	0.882498	0.326069
Kurtosis	2.006379	0.566834	− 0.93908	− 1.17566
Skewness	− 1.32916	− 0.76632	− 0.35171	− 0.02032
Range	5	5	3.6774	2.263446
Minimum	0	0	1.3226	2.358789
Maximum	5	5	5	4.622235
Sum	538.3791	559.2627	542.772	534.2143
Total samples	152	152	152	152

**Table 7:** Descriptive statistics of academic performances of undergraduates studying Banking and Finance.

	Grade Point Average (GPA)			
	First semester	Second semester	First semester	Aggregate
	(2017)	(2016)	(2016)	
Mean	3.411659	3.398917	3.062883	3.566969
Standard Error	0.151408	0.155527	0.173624	0.021297
Median	3.3913	3.375	2.8182	3.556119
Mode	4.619	3.375	–	–
Standard Deviation	0.815356	0.837536	0.934994	0.11469
Sample Variance	0.664806	0.701467	0.874214	0.013154
Kurtosis	0.073257	− 0.73148	− 0.68643	− 0.12852
Skewness	− 0.16815	0.122718	0.38637	− 0.02051
Range	3.4048	3.0729	3.5584	0.477883
Minimum	1.5	1.7692	1.2333	3.309419
Maximum	4.9048	4.8421	4.7917	3.787302
Sum	98.9381	98.5686	88.8236	103.4421
Total samples	29	29	29	29

**Table 8:** Descriptive statistics of academic performances of undergraduates studying Business Administration.

	Grade Point Average (GPA)			
	First semester	Second semester	First semester	Aggregate
	(2017)	(2016)	(2016)	
Mean	3.368239	3.532943	3.036686	3.376434
Standard Error	0.093754	0.088038	0.109293	0.039928
Median	3.48075	3.5625	3.0801	3.344223
Mode	3.7308	4.125	3.2917	–
Standard Deviation	0.859267	0.806881	1.001691	0.365944
Sample Variance	0.73834	0.651056	1.003385	0.133915
Kurtosis	0.429708	− 0.7147	− 0.85686	0.066188
Skewness	− 0.55211	− 0.19753	− 0.14478	0.197826
Range	4.1429	3.2857	4.3182	1.77455
Minimum	0.8571	1.7143	0.5	2.577491
Maximum	5	5	4.8182	4.352041
Sum	282.9321	296.7672	255.0816	283.6204
Total samples	84	84	84	84

**Table 9:** Descriptive statistics of academic performances of undergraduates studying Economics.

	Grade Point Average (GPA)			
	First semester	Second semester	First semester	Aggregate
	(2017)	(2016)	(2016)	
Mean	3.565416	3.531844	3.346186	3.396855
Standard Error	0.096568	0.091972	0.110489	0.048397
Median	3.67425	3.58335	3.4792	3.428324
Mode	4.5	4.1667	4.52	–
Standard Deviation	0.936257	0.891702	1.071234	0.469223
Sample Variance	0.876578	0.795132	1.147543	0.22017
Kurtosis	− 0.381	− 1.07088	− 0.97002	− 0.45452
Skewness	− 0.5932	− 0.23577	− 0.34232	0.21792
Range	4	3.2367	4	1.974028
Minimum	1	1.68	1	2.491155
Maximum	5	4.9167	5	4.465183
Sum	335.1491	331.9933	314.5415	319.3044
Total samples	94	94	94	94

**Table 10:** Descriptive statistics of academic performances of undergraduates studying Sociology.

	Grade Point Average (GPA)			
	First semester	Second semester	First semester	Aggregate
	(2017)	(2016)	(2016)	
Mean	3.350137	3.395533	3.193853	3.309902
Standard Error	0.117221	0.099187	0.110424	0.029326
Median	3.4348	3.4783	3.2	3.403728
Mode	3.7391	3.75	4	–
Standard Deviation	0.837129	0.708339	0.788583	0.209426
Sample Variance	0.700784	0.501744	0.621863	0.043859
Kurtosis	1.215688	− 0.28199	− 0.12678	− 0.92206
Skewness	− 0.74502	− 0.29371	− 0.50226	− 0.59545
Range	4.3286	3.3043	3.5417	0.787557
Minimum	0.5714	1.6957	1.2083	2.846396
Maximum	4.9	5	4.75	3.633954
Sum	170.857	173.1722	162.8865	168.805
Total samples	51	51	51	51

**Table 11:** Descriptive statistics of academic performances of undergraduates studying International Relations.

	Grade Point Average (GPA)			
	First semester	Second semester	First semester	Aggregate
	(2017)	(2016)	(2016)	
Mean	3.209772	3.243783	3.188024	3.196433
Standard Error	0.092809	0.071523	0.070455	0.030202
Median	3.32665	3.31625	3.2404	3.206606
Mode	4.25	3.7826	4.5	#N/A
Standard Deviation	0.899817	0.693437	0.683089	0.29282
Sample Variance	0.80967	0.480855	0.466611	0.085743
Kurtosis	1.585275	− 0.20814	0.688421	− 0.54124
Skewness	− 1.00579	− 0.29734	− 0.37832	0.158999
Range	4.7	3.2795	3.6852	1.246327
Minimum	0	1.2857	0.8148	2.639584
Maximum	4.7	4.5652	4.5	3.885911
Sum	301.7186	304.9156	299.6743	300.4647
Total samples	94	94	94	94

**Table 12:** Descriptive statistics of academic performances of undergraduates studying Political Science.

	Grade Point Average (GPA)			
	First semester	Second semester	First semester	Aggregate
	(2017)	(2016)	(2016)	
Mean	3.09921	3.079923	3.091737	3.122432
Standard Error	0.102475	0.086062	0.084182	0.039827
Median	3.2609	3.0435	2.963	3.126778
Mode	3.3913	3	3.7083	–
Standard Deviation	0.875545	0.735312	0.719247	0.340283
Sample Variance	0.766579	0.540684	0.517317	0.115793
Kurtosis	1.468504	− 0.54403	− 0.77399	− 1.1565
Skewness	− 0.67475	− 0.05645	0.311734	− 0.15438
Range	4.8571	3.0957	3	1.210965
Minimum	0	1.6	1.7917	2.51653
Maximum	4.8571	4.6957	4.7917	3.727494
Sum	226.2423	224.8344	225.6968	227.9376
Total samples	73	73	73	73

**Table 13:** Descriptive statistics of academic performances of undergraduates studying Agricultural Engineering.

	Grade Point Average (GPA)			
	First semester	Second semester	First semester	Aggregate
	(2017)	(2016)	(2016)	
Mean	3.478852	3.473233	3.172098	3.381699
Standard Error	0.14127	0.122875	0.123122	0.035197
Median	3.58125	3.58335	3.4	3.375671
Mode	4.44	4.0833	3.4	–
Standard Deviation	0.978745	0.851305	0.853014	0.24385
Sample Variance	0.957941	0.72472	0.727634	0.059463
Kurtosis	1.082866	− 0.56261	− 1.02563	0.124383
Skewness	− 0.81934	− 0.47954	− 0.37219	0.56286
Range	4.64	3.2584	2.9917	1.051886
Minimum	0.2	1.5333	1.56	2.993714
Maximum	4.84	4.7917	4.5517	4.0456
Sum	166.9849	166.7152	152.2607	162.3215
Total samples	48	48	48	48

**Table 14:** Descriptive statistics of academic performances of undergraduates studying Chemical Engineering.

	Grade Point Average (GPA)			
	First semester	Second semester	First semester	Aggregate
	(2017)	(2016)	(2016)	
Mean	3.517811	3.421146	3.27336	3.233792
Standard Error	0.113916	0.106236	0.102236	0.050809
Median	3.64275	3.4375	3.1574	3.295881
Mode	4.6087	3.25	3	–
Standard Deviation	0.953093	0.888836	0.855369	0.425101
Sample Variance	0.908387	0.790029	0.731657	0.180711
Kurtosis	− 0.11782	− 0.5939	− 0.78012	− 1.21276
Skewness	− 0.5707	− 0.4071	− 0.06581	− 0.2729
Range	4.3846	3.5333	3.5083	1.410907
Minimum	0.6154	1.4667	1.2	2.444588
Maximum	5	5	4.7083	3.855494
Sum	246.2468	239.4802	229.1352	226.3655
Total samples	70	70	70	70

**Table 15:** Descriptive statistics of academic performances of undergraduates studying Civil Engineering.

	Grade Point Average (GPA)			
	First semester	Second semester	First semester	Aggregate
	(2017)	(2016)	(2016)	
Mean	3.521384	3.302868	3.181576	3.292949
Standard Error	0.083006	0.081553	0.073192	0.04283
Median	3.68	3.30515	3.19675	3.29454
Mode	4.76	3	3	–
Standard Deviation	0.989135	0.971813	0.872189	0.510381
Sample Variance	0.978389	0.944421	0.760714	0.260489
Kurtosis	0.824365	− 0.35277	− 0.30143	− 1.17186
Skewness	− 0.81698	− 0.41172	− 0.14492	− 0.02171
Range	5	4.5357	4.1877	1.804352
Minimum	0	0.4643	0.6923	2.424246
Maximum	5	5	4.88	4.228598
Sum	500.0365	469.0072	451.7838	467.5988
Total samples	142	142	142	142

**Table 16:** Descriptive statistics of academic performances of undergraduates studying Electrical and Information Engineering.

	Grade Point Average (GPA)			
	First semester	Second semester	First semester	Aggregate
	(2017)	(2016)	(2016)	
Mean	3.234168	3.134438	3.348105	3.190894
Standard Error	0.093358	0.07606	0.068441	0.048762
Median	3.3704	3.1786	3.2667	3.169778
Mode	4	3.5	3	–
Standard Deviation	1.227933	1.00041	0.9002	0.641366
Sample Variance	1.50782	1.00082	0.81036	0.41135
Kurtosis	0.386574	− 0.50304	− 0.50456	− 0.29363
Skewness	− 0.85019	− 0.24748	− 0.13679	− 0.23311
Range	5	4.3688	4.1667	3.288419
Minimum	0	0.6	0.8333	1.1212
Maximum	5	4.9688	5	4.409619
Sum	559.5111	542.2578	579.2221	552.0247
Total samples	173	173	173	173

**Table 17:** Descriptive statistics of academic performances of undergraduates studying Mechanical Engineering.

	Grade Point Average (GPA)			
	First semester	Second semester	First semester	Aggregate
	(2017)	(2016)	(2016)	
Mean	3.464989	3.325181	3.297984	3.258349
Standard Error	0.0711	0.071058	0.059607	0.042862
Median	3.52	3.2667	3.32	3.293257
Mode	4	3.1667	3	–
Standard Deviation	0.907746	0.907213	0.761006	0.547231
Sample Variance	0.824003	0.823036	0.57913	0.299461
Kurtosis	0.487968	− 0.41704	− 0.11727	− 0.36929
Skewness	− 0.70829	− 0.31852	− 0.27276	− 0.40715
Range	4.76	4.0769	3.3572	2.631019
Minimum	0.24	0.9231	1.4828	1.651789
Maximum	5	5	4.84	4.282807
Sum	564.7932	542.0045	537.5714	531.1109
Total samples	163	163	163	163

**Table 18:** Descriptive statistics of academic performances of undergraduates studying Biochemistry.

	Grade Point Average (GPA)			
	First semester	Second semester	First semester	Aggregate
	(2017)	(2016)	(2016)	
Mean	3.415608	3.454415	3.38042	3.392544
Standard Error	0.140644	0.102213	0.109619	0.062291
Median	3.4375	3.4286	3.47915	3.511844
Mode	5	4	3.5833	–
Standard Deviation	1.08942	0.791735	0.849102	0.482507
Sample Variance	1.186836	0.626845	0.720975	0.232813
Kurtosis	0.006557	− 0.43395	− 0.88885	− 0.22328
Skewness	− 0.55988	− 0.16745	0.069715	− 0.68658
Range	4.7917	3.4028	3.0278	1.862878
Minimum	0.2083	1.5172	1.9722	2.341733
Maximum	5	4.92	5	4.204612
Sum	204.9365	207.2649	202.8252	203.5526
Total samples	60	60	60	60

**Table 19:** Descriptive statistics of academic performances of undergraduates studying Microbiology.

	Grade Point Average (GPA)			
	First semester	Second semester	First semester	Aggregate
	(2017)	(2016)	(2016)	
Mean	3.105357	3.133487	3.319841	3.186228
Standard Error	0.189018	0.148619	0.13522	0.148879
Median	3.3003	3.27715	3.27085	3.1532
Mode	0	3.6071	4.1667	–
Standard Deviation	1.281985	1.007984	0.917109	1.009744
Sample Variance	1.643487	1.016032	0.841089	1.019583
Kurtosis	0.503793	− 0.21781	− 0.79087	− 0.02491
Skewness	− 0.93608	− 0.46646	− 0.17788	− 0.5002
Range	5	4.1111	3.35	3.9784
Minimum	0	0.7037	1.5667	0.827167
Maximum	5	4.8148	4.9167	4.805567
Sum	142.8464	144.1404	152.7127	146.5665
Total samples	46	46	46	46

**Table 20:** Descriptive statistics of academic performances of undergraduates studying industrial Chemistry.

	Grade Point Average (GPA)			
	First semester	Second semester	First semester	Aggregate
	(2017)	(2016)	(2016)	
Mean	3.306633	2.914775	2.602633	3.081596
Standard Error	0.239512	0.226467	0.220035	0.022954
Median	3.24715	3.03675	2.51485	3.112263
Mode	–	2	–	–
Standard Deviation	0.829693	0.784505	0.762223	0.079515
Sample Variance	0.68839	0.615447	0.580983	0.006323
Kurtosis	− 0.49905	− 1.45085	− 1.3482	3.121076
Skewness	0.175735	0.022201	0.231653	− 2.06314
Range	2.72	2.2067	2.07	0.251976
Minimum	2	1.8333	1.65	2.887897
Maximum	4.72	4.04	3.72	3.139873
Sum	39.6796	34.9773	31.2316	36.97915
Total samples	12	12	12	12

**Table 21:** Descriptive statistics of academic performances of undergraduates studying Computer Science.

	Grade Point Average (GPA)			
	First semester	Second semester	First semester	Aggregate
	(2017)	(2016)	(2016)	
Mean	3.013887	2.948965	2.952319	2.862743
Standard Error	0.128438	0.110269	0.090685	0.04083
Median	3.08545	2.9565	2.9	2.838766
Mode	3.0526	2.9565	2.125	–
Standard Deviation	1.177157	1.010635	0.831137	0.374215
Sample Variance	1.385698	1.021382	0.690789	0.140037
Kurtosis	− 0.00489	− 0.0371	− 0.21249	− 0.44557
Skewness	− 0.56733	− 0.32649	0.093098	0.183183
Range	5	4.591	3.9028	1.592913
Minimum	0	0.1481	0.8889	2.083531
Maximum	5	4.7391	4.7917	3.676444
Sum	253.1665	247.7131	247.9948	240.4704
Total samples	84	84	84	84

**Table 22:** Descriptive statistics of academic performances of undergraduates studying Mathematics.

	Grade Point Average (GPA)			
	First semester	Second semester	First semester	Aggregate
	(2017)	(2016)	(2016)	
Mean	2.50275	2.3908	2.04795	2.588869
Standard Error	0.621084	0.438643	0.446336	0.068625
Median	2.4472	2.1868	2.24165	2.654247
Mode	–	–	–	–
Standard Deviation	1.242167	0.877286	0.892671	0.137251
Sample Variance	1.542979	0.769631	0.796862	0.018838
Kurtosis	1.206473	2.454761	− 2.3235	3.976493
Skewness	0.261558	1.293645	− 0.65725	− 1.9928
Range	3.0214	2.0696	1.8567	0.28075
Minimum	1.0476	1.56	0.9259	2.383117
Maximum	4.069	3.6296	2.7826	2.663867
Sum	10.011	9.5632	8.1918	10.35548
Total samples	4	4	4	4

**Table 23:** Descriptive statistics of academic performances of undergraduates studying Physics.

	Grade Point Average (GPA)			
	First semester	Second semester	First semester	Aggregate
	(2017)	(2016)	(2016)	
Mean	3.38375	2.59433	2.42551	2.873113
Standard Error	0.160386	0.199168	0.249894	0.043127
Median	3.21895	2.58105	2.1777	2.851922
Mode	3	2.3704	–	–
Standard Deviation	0.507186	0.629823	0.790233	0.136378
Sample Variance	0.257237	0.396678	0.624468	0.018599
Kurtosis	1.069219	0.977881	− 0.40948	2.213211
Skewness	1.472281	− 0.0355	0.76557	1.018483
Range	1.4643	2.3269	2.3334	0.503328
Minimum	2.9643	1.4231	1.619	2.673289
Maximum	4.4286	3.75	3.9524	3.176617
Sum	33.8375	25.9433	24.2551	28.73113
Total samples	10	10	10	10

## 2 Experimental design, materials and methods

The cumulative GPA of the sample population of students for the semester highlighted was obtained from the Centre for Systems and Information Services Units of the University. Motivated by the need to promote evidence-based research in academic excellence, a spread-sheet containing the detailed datasets is attached to this article. The descriptive statistics and frequency distributions of academic performance data are presented in with the use of tables and graphs to ease the description of the data.

## 3 Data exploration

### 3.1 Overall aggregated Grade Point Average by semesters

**Fig. 1:**
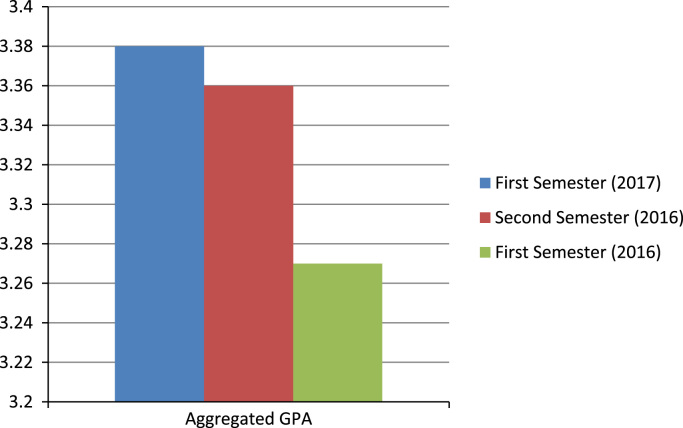
Variation of GPA in the three semesters considered of undergraduates in all colleges.

Fig. 1 show the highest aggregated GPA was recorded in the 2017 first semester, followed by 2016 s semester and then 2016 first semester.

### 3.2 Aggregated Grade Point Average by levels in the colleges

**Fig. 2:**
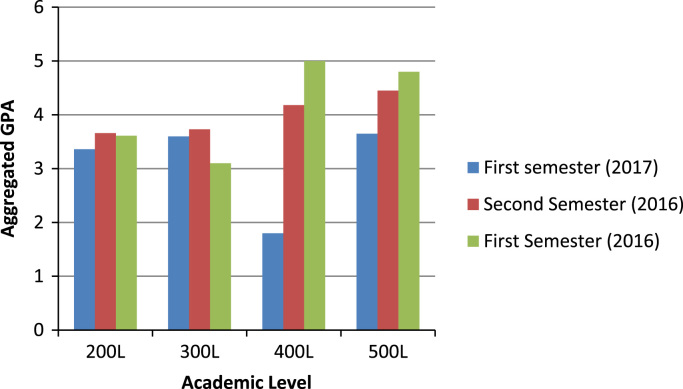
Summary of all aggregated GPA across levels in the college of Agricultural Sciences.

The section contains the description of aggregated GPA by colleges. Fig. 2 shows the academic performances of undergraduates in the college of Agricultural Sciences (CAS). More specifically, the figures show that 200 level students in CAS performed best in 2016 s semester than the other semesters, while 300 level students performed best in second semester 2016 compared with their performance in the two other semesters. Furthermore, the 400 level in CAS performed best in 2016 first semester, and the first semester 2016 was the best for the 500L students of the college.

**Fig. 3:**
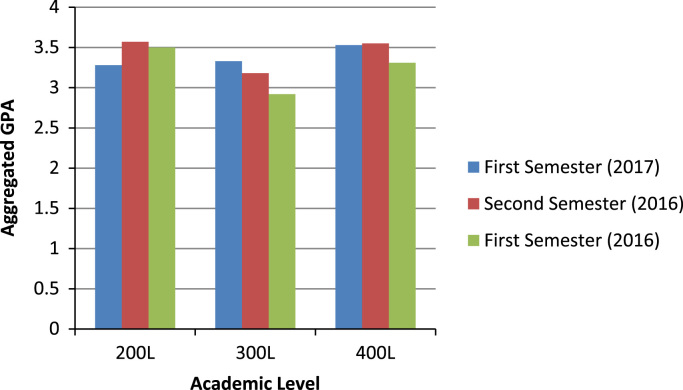
Summary of all Aggregated GPA across levels in the college of Business and Social Sciences.

The description of the academic performances of undergraduates by level in the college of Business and Social Sciences (CBSS) are captured in Fig. 3. Fig. 3 revealed that the aggregated GPA of 200 levels was highest in the second semester of 2016. In the same vein, the 300 levels of the CBSS performed best in the first semester of 2017 than in the other two semesters, while the highest aggregated GPA for 400L students was in the second semester of 2016.

**Fig. 4:**
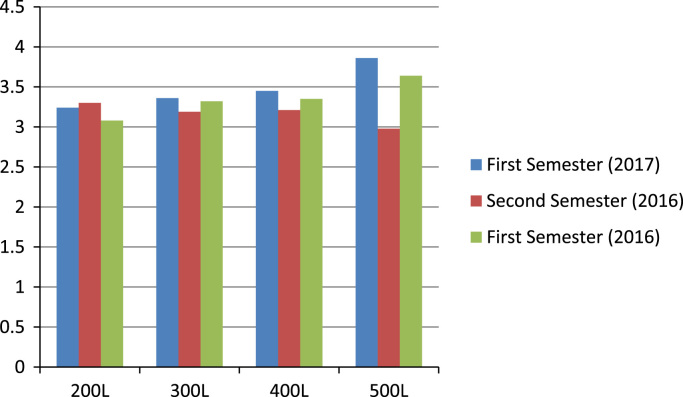
Summary of all aggregated GPA across levels in the college of Science and Engineering.

Fig. 4 depicts the academic performances of undergraduates in the college of Science and Engineering (CSE). The analysis of the academic performance based on Fig. 4 shows that 200 levels students in CSE performed best in 2016 s semester, while the 300 level students had the highest aggregated GPA in the first semester of 2017. In addition, students in the 400 level and 500 levels performed best in the first semester of 2017.

### 3.3 Aggregated Grade Point Average by programmes in the colleges

**Fig. 5:**
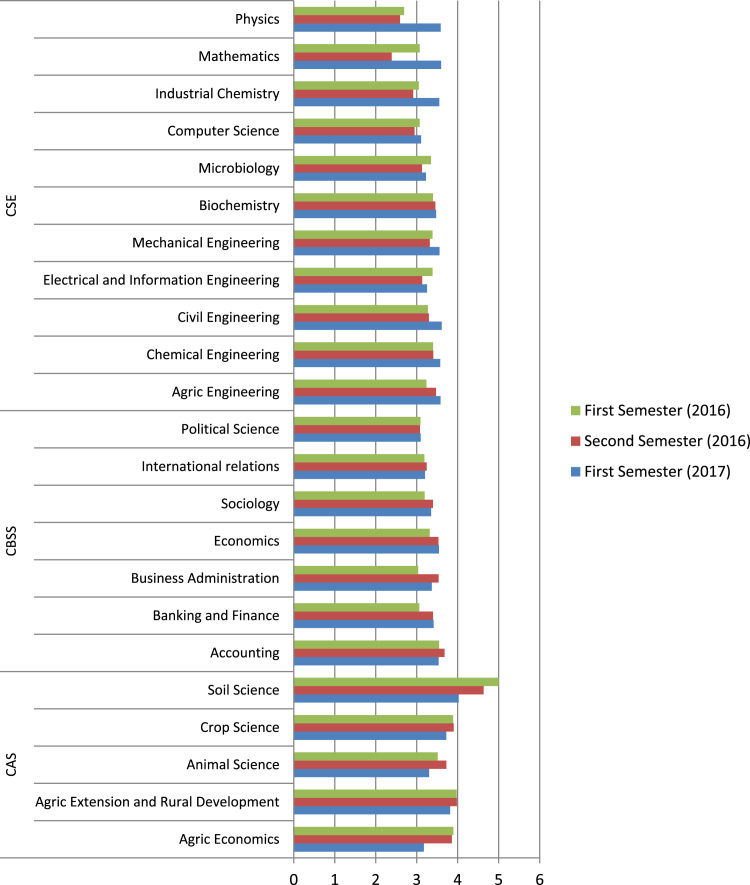
Semester academic performances in programmes in the Colleges.

Fig. 5 shows the description of the comprehensive academic performances of the programmes across the three major colleges in the semesters under review. The data show that these programmes- Soil Science, Agricultural Extension and Rural Development, International Relations, Computer Science and Political Science, had the least aggregated GPA figures as arranged in descending order.

Moreover, Soil Science, Agricultural Extension & Rural Development and Crop Science programmes recorded the highest aggregated GPA in the second semester of 2016; Industrial Chemistry, Physics and Mathematics programmes in the CSE had the least aggregated GPA. Generally, students in the programmes offered in the College of Agricultural Sciences had the highest aggregated GPA in the semesters under review.

### 3.4 Aggregated GPA by colleges

**Fig. 6:**
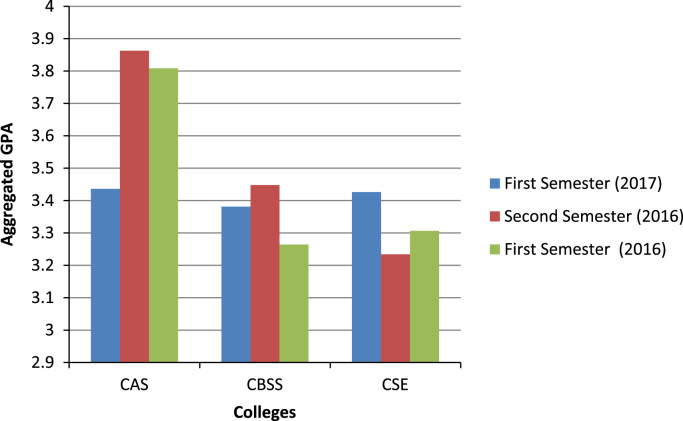
The students’ performances by college per semester.

As shown in Fig. 6, the colleges of Agricultural Sciences and Business and Social Sciences recorded the highest aggregated GPA in the second semester of 2016. Although the college of Science and Engineering recorded the worst aggregated GPA in the second semester of 2016, it had that the highest aggregated GPA in first semester of 2017.

### 3.5 Aggregated GPA for the three semesters combined

**Fig. 7:**
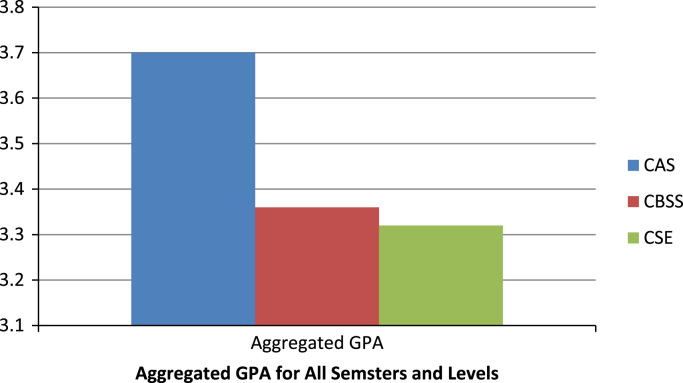
The overall students’ performance by college across the three semesters (aggregated GPA).

The description of the academic performances of the colleges in terms of the overall aggregated GPA that computes the GPA across the three semesters under review shows that the students in the college of Agricultural Sciences considerably performed best academically, followed by the college of Business and Social Sciences. The students of the college of Science and Engineering had the worse academic performances (Fig. 7).
